# Inhalation of *Pelargonium graveolens* Essential Oil Alleviates Pain and Related Anxiety and Stress in Patients with Lumbar Spinal Stenosis and Moderate to Severe Pain

**DOI:** 10.3390/ph17010001

**Published:** 2023-12-19

**Authors:** Eunhye Seo, Yoonah Cho, Jeong-Min Lee, Geun Hee Seol

**Affiliations:** 1 Department of Basic Nursing Science, College of Nursing, Korea University, Seoul 02841, Republic of Korea; 2 KT&G Central Research Institute, Daejeon 34337, Republic of Korea; 3 BK21 FOUR Program of Transdisciplinary Major in Learning Health Systems, Graduate School, Korea University, Seoul 02841, Republic of Korea

**Keywords:** anxiety, geranium essential oil, lumbar spinal stenosis, pain, stress

## Abstract

Pain in lumbar spinal stenosis (LSS) patients is closely associated with psychological factors, including anxiety, stress, and depression, and is a critical determinant of patient daily functionality and overall quality of life. The present study evaluated the effects of inhalation of *Pelargonium graveolens* (geranium) essential oil (GEO) on pain and related psychological factors in LSS patients. Fifty-nine patients, categorized as having mild or moderate to severe pain based on pain visual analog scale (VAS) scores, were randomly assigned to inhalation of 1% GEO or placebo control (PC). No significant differences between GEO and PC were observed in patients with mild pain, whereas differences in anxiety-VAS and stress-VAS scores were observed in patients with moderate to severe pain. Anxiety-VAS and stress-VAS scores decreased significantly after GEO but not after PC inhalation. Regardless of the severity of pain, post-intervention pain-VAS scores were significantly lower in the GEO group than in the PC group. In summary, GEO reduced pain and improved anxiety and stress, particularly among patients with moderate to severe pain. These findings suggest that GEO inhalation may have potential as an adjunct therapy for improving pain management and alleviating anxiety and stress in LSS patients with insufficient responses to pharmacological pain control.

## 1. Introduction

Lumbar spinal stenosis (LSS) is a condition marked by persistent pain in the lower back and legs due to compression of the central canal or nerve roots, a compression resulting from degenerative processes such as reduced intervertebral disc height, bony outgrowths, and hypertrophy of the ligamentum flavum [1]. Analysis of data from the Healthcare Bigdata Hub and the Korean Statistical Information Service between 2012 and 2016 indicated that the incidence of LSS was highest in patients aged 60 years and older and that LSS treatment incurred high medical expenses, such that LSS was the costliest spinal disorder during this study period [2]. The typical symptoms experienced by LSS patients, such as lower back pain and neurogenic claudication while walking, restrict their daily activities, notably affecting their walking capacity and reducing their quality of life [3]. Despite radiological assessments showing evidence of neural constriction in the lumbar spinal canal and neural foramina, treatment is generally not recommended in the absence of accompanying pain and impaired physical function [4]. Rather, current medical guidelines recommend conservative treatment, including medication for pain control, physical therapy, and rehabilitation, as the initial treatment for symptomatic LSS patients. Surgery may be considered when pain and impaired physical function do not respond to conservative methods or for patients with exacerbated acute neurological symptoms [5]. That is, the management of patients with LSS is tailored to the degree of pain experienced, with the primary goal being the prevention of physical functional impairments and the preservation of daily life functions through effective pain control [4,5].

The International Association for the Study of Pain has defined pain as "an unpleasant sensory and emotional experience associated with actual or potential tissue damage or described in terms of such damage” [6]. Thus, interventions for pain in LSS patients should consider not only the sensory components transmitted to the brain through neural pathways but also psychological factors related to pain, such as anxiety, depression, low self-efficacy, and fear of movement [7]. Medications for pain control include acetaminophen, nonsteroidal anti-inflammatory drugs (NSAIDs), opioids, and muscle relaxants, with anxiolytics and/or antidepressants considered for patients with severe psychological symptoms [5]. Drugs that affect the central nervous system, however, are often associated with adverse effects, including physical and psychological dependence. The high prevalence of LSS among elderly subjects increases concerns regarding the side effects of these drugs [8]. Hence, there is growing interest in alternative therapies for the management of pain in patients with LSS [9,10].

Aromatherapy is widely employed for pain management due to its potential for physiological and psychological improvements in the context of pain [11,12]. Recently, the inhalation of a mixture of ginger, lemongrass, and peppermint oils has been reported to have long- and short-term analgesic effects in elderly persons with osteoarthritis [13], and oral administration of ylang-ylang essential oil was found to alleviate pain and anxiety in animal models of neuropathic pain [14]. Additionally, the inhalation of bergamot essential oil has been reported to reduce chronic pain as well as lower heart rate and respiratory rate in post-laminectomy patients [15]. *Pelargonium graveolens* (geranium) essential oil (GEO), a natural substance widely included in products such as cosmetics and soaps, has been reported to exert effects on physiological and psychological factors related to pain [16]. *Pelargonium* species are currently the most common source of essential oils cultivated commercially, and studies on their regulatory and intervention roles in pathological processes contribute to increased clinical utility [17]. For example, GEO inhalation alleviated acute pain and reduced blood pressure and heart rate in patients undergoing colectomy [18], and decreased anxiety and emotional depression in patients with acute myocardial infarction [19]. GEO inhalation has also been shown to reduce fatigue among ICU nurses [20] and to mitigate edema and pain induced by albumin in mice [21]. These effects of GEO were associated with its impact on the hypothalamic-pituitary-adrenal axis, including its antioxidative properties and reductions in glucocorticoids [19,22]. To date, however, no studies have evaluated the effects of GEO on pain and related psychological factors in LSS patients with chronic pain. Because pain severity has a direct impact on quality of life in LSS patients [1,23], the present study was designed to differentiate and validate the effects of GEO inhalation based on the severity of pain.

## 2. Results

### 2.1. Chemical Profile of Geranium Essential Oil

Gas chromatography/mass spectrometry (GC-MS) analysis of the composition of GEO identified a total of 24 components, accounting for 99.9% of its overall composition. Citronellol (26.43%) was the most abundant compound, followed by geraniol (12.52%), citronellyl formate (8.78%), γ-eudesmol (8.45%), isomenthone (7.54%), linalool (5.59%), geranyl formate (3.76%), l-menthone (2.96%), geranyl tiglate (2.63%), (−)-β-cadinene (2.45%), and geranyl butyrate (2.23%). Caryophyllene, cis-rose oxide, citral, (−)-β-bourbonene, and citronellyl butyrate each accounted for 1–2% of the composition of GEO, and β-eudesmol, α-agarofuran, geranyl acetate, and α-pinene for less than 1% each (Table 1).

### 2.2. Baseline Characteristics of LSS Patients as a Function of Patient-Reported Pain Severity

A total of 57 patients were analyzed, consisting of 15 (26.4%) men and 42 (73.6%) women, with a mean (standard deviation, SD) age of 65.75 (7.78) years. Of these 57 patients, 16 (28.1%) had mild pain and 41 (71.9%) had moderate to severe pain, with mean (SD) pain-VAS scores of 2.34 (0.69) and 6.10 (1.76), respectively (*p* < 0.001). Age, sex, smoking and alcohol history, and the presence of hypertension did not differ significantly between the groups of patients with mild, moderate, or severe pain (Table 2). Medications used for pain control included NSAIDs, opioids, and pregabalin, with acetaminophen or ibuprofen being used in conjunction with other medications. The frequency of use of each pain medication did not differ significantly between patients with mild and moderate to severe pain, and there were also no significant between-group differences in sBP, dBP, HR, and serum biochemical parameters. However, GAD-7 scores (*p* = 0.035), anxiety-VAS scores (*p* = 0.001), stress-VAS scores (*p* = 0.004), and depression-VAS scores (*p* = 0.005) were significantly higher in patients with moderate to severe pain than in patients with mild pain (Table 2).

### 2.3. Effects of Geranium Essential Oil Inhalation on Anxiety-VAS, Stress-VAS, and Depression-VAS Scores as a Function of Pain Severity

Patients were randomly assigned to receive almond oil (placebo control, PC) or GEO based on pain severity. Prior to inhalation, the baseline characteristics of these groups, including age, gender, types of pain control medications, sBP, dBP, and HR, did not differ significantly. In addition, anxiety-VAS scores, stress-VAS scores, and depression-VAS scores did not differ significantly in patients assigned to the PC and GEO groups (Table 3).

The impact of GEO inhalation was assessed separately in the mild pain group and the moderate-to-severe pain group. Psychological factors associated with pain were assessed before and after intervention in the individual PC and GEO groups, as well as being compared in the two groups. Within the mild pain group, there were no significant differences observed between patients who received PC and GEO. However, stress-VAS scores in patients with mild pain were significantly reduced following inhalation of both PC (z = −0.24, *p* = 0.039) and GEO (z = −1.62, *p* = 0.043). 

In patients with moderate to severe pain, significant differences between the PC and GEO groups were observed in anxiety-VAS scores (*p* = 0.002) and stress-VAS scores (*p* = 0.003). Anxiety-VAS scores (z = −1.08, *p* = 0.002), stress-VAS scores (z = −2.08, *p* < 0.001), and depression-VAS scores (z = −0.63, *p* = 0.030) were significantly lower after than before GEO inhalation but did not differ significantly before and after PC inhalation in patients with moderate to severe pain (Table 4).

### 2.4. Pain-Related Variables at Baseline and after Geranium Essential Oil or Placebo Control Inhalation

The effects of GEO inhalation on variables related to physical and psychological pain were also evaluated in all patients, regardless of self-reported pain severity. At baseline, there were no significant differences between the PC and GEO groups in pain-VAS scores, anxiety-VAS scores, stress-VAS scores, and depression-VAS scores, or in sBP, dBP, HR, and other general characteristics. Following PC inhalation, none of the variables differed significantly, except for dBP (z = 1.50, *p* = 0.012). Following GEO inhalation, significant reductions were observed in pain-VAS scores (z = −1.41, *p* < 0.001), anxiety-VAS scores (z = −0.88, *p* = 0.001), stress-VAS scores (z = −1.93, *p* < 0.001), depression-VAS scores (z = −0.79, *p* = 0.006), and sBP (z = −5.24, *p* = 0.002). When the two groups were compared, pain-VAS scores (*p* = 0.003), anxiety-VAS scores (*p* = 0.004), and stress-VAS scores (*p* = 0.001) were significantly lower in patients assigned to GEO than to PC inhalation (Table 5).

## 3. Discussion

The present study evaluated the effects of GEO inhalation on anxiety, stress, and depression in patients with LSS classified as having mild, moderate, or severe pain. GEO inhalation reduced anxiety and stress in patients with moderate to severe pain, suggesting that inhalation of GEO may reduce the occurrence of anxiety and depressive disorders caused by poorly controlled pain. These effects may be due to both a reduction in pain perception by patients with LSS and enhanced psychological stabilization, particularly among patients with pronounced pain severity.

The persistent pain in LSS patients is significantly associated with impaired physical function, including walking ability, and reduced quality of life [24,25]. Furthermore, pain has been found to exacerbate psychological factors such as anxiety, stress, and depression, leading to increased fear and avoidance of physical activities, ultimately resulting in physical disabilities and social and economic isolation [3,26]. The present study found that anxiety, stress, and depression levels were greater in LSS patients with moderate to severe pain than with mild pain. Therefore, LSS patients with higher pain severity require more attention and care concerning both physical discomfort and psychological instability [7,27].

GEO inhalation may result in comprehensive improvements in pain and related psychological and emotional factors experienced by LSS patients. GEO has been reported to possess anti-inflammatory and analgesic effects through the regulation of inflammatory mediators [28,29]. A recent review in the field of dentistry emphasized that the anti-inflammatory properties of GEO could potentially be exploited in oral applications [30]. In animal experiments, application of GEO effectively alleviated egg-albumin-induced hind paw edema and pain, with no signs of toxicity or alterations in general behavior or other physical activities [31]. GEO was found to reduce inflammatory mediators, such as interleukin (IL)-1β and tumor necrosis factor (TNF)-α, thereby inhibiting the expression of cyclooxygenase-2 (COX-2), in THP-1 macrophages with lipopolysaccharide (LPS)-induced inflammation [28]. Citronellol and geraniol, which make up about 40% of the contents of GEO, inhibited the production of prostaglandin E_2_ (PGE_2_) and the expression of COX-2 in RAW 264.7 macrophages with LPS-induced inflammation [29]. NSAIDs are the preferred primary medications for relieving pain and alleviating associated physical impairments in patients with LSS, primarily due to the ability of NSAIDs to inhibit PGE_2_ production and COX-2 expression [5]. However, long-term use of NSAIDs not only increases the incidence of side effects, including upper abdominal discomfort, but is contraindicated in many elderly patients who are unable to tolerate NSAIDs due to conditions such as chronic kidney disease [31]. Thus, GEO may have potential benefits for these patients, offering anti-inflammatory and analgesic effects similar to NSAIDs but with reduced side effects [32].

Intraperitoneal administration of GEO has been found to reduce the duration of immobility during forced swimming tests in male Swiss albino mice [33]. This effect may be counteracted by a 5-HT1A receptor antagonist, suggesting that components of GEO may act as direct or indirect serotonin receptor agonists [33]. The effects associated with serotonin are also related to pain control. Duloxetine, an antidepressant that acts as a serotonin-norepinephrine reuptake inhibitor, has been found to alleviate neuropathy by enhancing the inhibitory pain pathway [34] and is effective in reducing chronic lower back pain in patients with LSS [35,36]. These findings suggest that GEO may improve pain perception and psychological factors related to pain, such as anxiety and stress, in LSS patients by acting as a serotonin receptor agonist and by inhibiting PGE_2_ production and COX-2 expression.

Neuropathic pain, characterized by its severity and difficulty of management, increases the incidence of anxiety and depression in LSS patients, significantly reducing their quality of life [37]. Although this pain is frequently treated with gabapentinoids, such as pregabalin and gabapentin [38], there is insufficient evidence regarding their efficacy and safety in relation to central nervous system inhibition [39]. The relationship between neuropathic pain and COX-2 has been evaluated in an LPS-induced neuropathic animal model [40], and a recent randomized controlled trial reported that NSAIDs can improve peripheral neuropathic pain in LSS patients [41]. Therefore, GEO may act as a long-term modulator of neuropathic pain in patients with LSS, offering potential advantages over NSAIDs, which are recommended only for short-term use.

COX-2 inhibition can prevent hypertrophy of the yellow ligament, which exacerbates spinal canal stenosis [42]. One of the major causes of hypertrophy of the yellow ligament is the accumulation of fibrosis due to chronic and repetitive inflammatory responses [43]. Inhibition of COX-2 expression in vascular endothelial cells can prevent hypertrophy of the yellow ligament, thereby delaying the progression of degenerative changes [43]. Although patients in this study were subjected to a single session of GEO inhalation, the inhibition of COX-2 expression following the long-term use of GEO may not only alleviate pain associated with physical and psychological factors but also prevent degenerative changes in the yellow ligament, ultimately improving the course of LSS.

Our findings indicate that GEO inhalation was effective in lowering perceived pain, anxiety, and stress in LSS patients with moderate to severe pain. These results provide additional evidence of the clinical effectiveness of GEO inhalation as a perceived pain control intervention in LSS patients with moderate to severe pain. Future work should assess the relative clinical benefits of GEO inhalation and psychological therapy in enhancing perceived quality of life in patients with LSS, a condition that poses risks to gait ability, physical function, and social and economic wellbeing due to worsening pain and related psychological aspects.

## 4. Materials and Methods

### 4.1. Participants and Study Design

**Figure 1:**
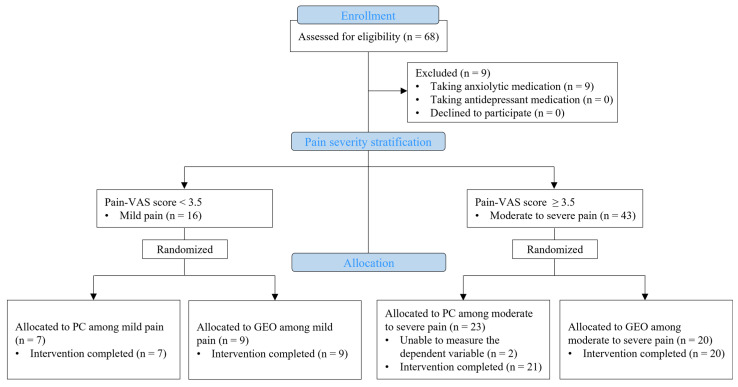
Patient flowchart according to the CONSORT statement for the report of randomized trials. Abbreviations: GEO, geranium essential oil inhalation group; PC, placebo control group; VAS, visual analog scale.

This randomized, controlled, pre- and post-experimental study was performed to assess the impact of GEO inhalation on pain and the associated physiological and psychological factors in LSS. This study was approved by the institutional review board of Korea University Guro Hospital (2022GR0183), and the clinical trial protocol was retrospectively registered with the Korean Clinical Research Information Service (KCT0008853). This study included patients diagnosed with LSS who received outpatient medical therapy in the neurosurgery department of Korea University Hospital from May 2022 to February 2023 and who provided informed consent to participate. Patients were excluded if they were unable to communicate, had significant medical history concerns, were currently undergoing hormone therapy or aromatherapy, or were taking medications for the treatment of anxiety or depression. Using the G*Power 3.1 program, it was estimated that a minimum of 41 individuals would be required to compare groups with a power of 0.50, an effect size of 0.40, and a significance level of 0.05 [44]. A total of 68 individuals expressed their willingness to participate; after screening based on selection and exclusion criteria, nine individuals currently taking anxiolytics were excluded. After completing the intervention, two individuals were unable to undergo the final measurement of the dependent variable and were also excluded, resulting in a final analysis of 57 individuals (Figure 1).

### 4.2. Randomization and Masking

An internal researcher was responsible for participant recruitment, screening, and the measurement of pre- and post-intervention variables, whereas external researchers were responsible for manufacturing the oil used for inhalation and for analyzing the data. External researchers prepared 100% almond oil (placebo control, PC) and 1% (*v*/*v*) GEO in almond oil, sealed the bottles, and delivered them to the internal researchers, who administered the inhalations to participants using sequentially numbered sealed tubes, ensuring that participants were allocated randomly.

### 4.3. GC/MS Spectrometry Profiling of Geranium Essential Oil

GEO was obtained from Aromarant Co. (Rottingen, Germany), and its constituent components were analyzed by GC/MS spectrometry with a capillary column (HP-INNOWAX; Agilent Technologies, Santa Clara, CA, USA). The carrier gas was helium at a flow rate of 1.0 mL/min, with the temperature maintained at 40 °C for 10 min and increased at a rate of 3 °C/min to 230 °C. Compounds were separated based on retention times and identified by comparing their mass spectra with those in the NIST05 2010 library (Supplementary Material Figure S1). Their homogeneity was confirmed by comparisons with reference compounds [45].

### 4.4. Classification Based on Self-Reported Pain Severity

The enrolled subjects were categorized into two groups based on pain severity, as measured by pain-VAS scores, into those with mild pain (n = 16) and moderate to severe pain (n = 41) (Figure 1). Subjects were asked to indicate the level of pain they experienced on a continuous horizontal line of 10 cm, with 0 cm indicating ‘no pain’ and 10 cm indicating ‘unbearable pain’. The measured values were classified into two groups using a cut-off point for the pain-VAS score based on research that employed hierarchical analysis of pain in patients with chronic musculoskeletal disease [46]. Subjects with pain-VAS scores ≤3.4 were categorized as having mild pain, whereas subjects with scores >3.5 were categorized as having moderate to severe pain [46].

### 4.5. Intervention

Participants stratified by pain severity were randomly allocated to inhale almond oil or 1% (*v*/*v*) GEO in almond oil. Anxiety-VAS scores, stress-VAS scores, and depression-VAS scores, along with BP and HR, were measured before and after a 20 min inhalation session. A 1% concentration of GEO was selected based on the preferences of ten healthy adults who evaluated 10%, 1%, and 0.1% GEO in almond oil, with 1% being preferred. 

During each inhalation session, participants were seated on comfortable chairs in a designated private area and exposed to the fragrance of almond oil or 1% geranium oil in almond oil applied to a 2 × 1 gauze and affixed to their philtrum area for 20 min. Immediately prior to the end of the inhalation, all participants were directed to take three deep breaths [45].

### 4.6. Outcome Measures

Anxiety-VAS scores, stress-VAS scores, and depression-VAS scores were measured as described above. Physiological factors related to pain, including sBP, dBP, and HR, were also measured. Baseline measurements were taken after participants were seated and remained still for 10 min. Post-intervention measurements were taken immediately after the intervention. The pre- and post-intervention sBP, dBP, and HR values reported represent the average of three measurements for each parameter, with intervals of 30 s to 1 min between measurements.

### 4.7. Statistics

The data were analyzed using SPSS 23.0 software (IBM SPSS, Inc., Chicago, IL, USA). The normality of continuous variables was confirmed using the Shapiro–Wilk test, with between-group comparisons determined using Mann–Whitney U tests. Categorical variables were assessed using the Chi-square test or Fisher’s exact test, as applicable. Group comparisons based on pre- and post-intervention measures, as well as pain severity, were performed using the Wilcoxon signed-rank test and Mann–Whitney U test. Statistical significance was determined at a 5% alpha level (95% confidence interval) with 80% power, and *p*-values <0.05 were considered statistically significant.

## 5. Conclusions

GEO reduced pain immediately after inhalation, as well as improving anxiety and stress in LSS patients with moderate to severe pain. These findings suggest the potential utility of GEO inhalation not only for pain relief but also for improving anxiety and stress-related factors. A strength of the present study is that a high proportion of participants were aged 60 years and older, making it representative of typical LSS patients. Moreover, we present notable distinctions in the efficacy of GEO inhalation depending on the severity of pain.

## Figures and Tables

**Table 1:** Chemical profile of geranium essential oil, as determined by GC/MS analysis.

No	RT (min)	Compound	Peak Area	% Area	No	RT (min)	Compound	Peak Area	% Area
1	34.576	Citronellol	994,299,533	26.43	13	17.831	cis-Rose oxide	66,694,494	1.77
2	37.328	Geraniol	470,930,376	12.52	14	32.995	Citral	66,694,494	1.77
3	29.079	Citronellyl formate	330,124,024	8.78	15	24.492	(−)-β-Bourbonene	66,141,115	1.76
4	45.323	γ-Eudesmol	317,871,828	8.45	16	35.677	Citronellyl butyrate	55,400,776	1.47
5	23.441	Isomenthone	283,628,500	7.54	17	31.873	Germacrene D	53,899,475	1.43
6	26.264	Linalool	210,448,432	5.59	18	48.110	2-Phenylethyl tiglate	53,393,219	1.42
7	32.212	Geranyl formate	141,407,662	3.76	19	36.326	Geranyl propionate	42,799,625	1.14
8	22.284	l-Menthone	111,436,296	2.96	20	49.084	β-Eudesmol	37,278,965	0.99
9	45.951	Geranyl tiglate	98,880,842	2.63	21	38.175	α-Agarofuran	31,336,257	0.83
10	33.765	(−)-β-Cadinene	92,330,860	2.45	22	36.488	Calamenene	30,803,550	0.82
11	38.909	Geranyl butyrate	83,822,233	2.23	23	34.082	Geranyl acetate	29,143,116	0.77
12	27.625	Caryophyllene	67,260,209	1.79	24	5.271	α-Pinene	26,062,379	0.69

Abbreviation: RT, retention time.

**Table 2:** Baseline demographic and clinical characteristics of patients with LSS as a function of patient-reported pain severity.

Variable	Mild Pain (n = 16)	Moderate to Severe Pain (n = 41)	*p*-Value
Pain-VAS, cm	2.34 ± 0.69	6.10 ± 1.76	>0.001 ***
Gender, women, n (%)	10 (62.50)	32 (78.05)	0.317 ^a^
Age, yr	64.44 ± 8.76	66.27 ± 7.41	0.539
BMI, kg/m^2^	25.49 ± 2.53	25.40 ± 3.39	0.118
Cigarette smoker, n (%)	2 (12.50)	6 (14.63)	1.000 ^a^
Alcohol drinker, n (%)	4 (25)	8 (19.51)	0.723 ^a^
Medications for pain control			
NSAIDs, n (%)	12 (75.00)	28 (68.29)	0.753 ^a^
Opioids, n (%)	3 (18.75)	14 (34.15)	0.342 ^a^
Pregabalin, n (%)	5 (31.25)	11 (26.83)	0.752 ^a^
Psychological status			
GAD-7, point	5.38 ± 4.95	8.49 ± 5.42	0.035 *
PHQ-9, point	7.31 ± 4.81	9.85 ± 6.30	0.155
Anxiety-VAS, cm	1.38 ± 1.51	3.98 ± 2.85	0.001 **
Stress-VAS, cm	2.13 ± 2.22	4.73 ± 3.10	0.004 **
Depression-VAS, cm	1.89 ± 1.85	3.96 ± 2.70	0.005 **
Hypertension, n (%)	9 (56.25)	23 (56.10)	1.000
sBP, mmHg	130.50 ± 11.78	130.12 ± 12.03	0.880
dBP, mmHg	73.63 ± 5.58	74.66 ± 11.52	0.852
HR, bpm/min	78.50 ± 14.29	78.83 ± 11.81	0.763
Serum biochemical parameters			
WBC count, μL	7181.25 ± 2293.82	6648.78 ± 1739.41	0.384
CRP, mg/dL	1.09 ± 1.23	1.25 ± 1.27	0.267
Total Ca^2+^, mg/dL	10.06 ± 0.60	9.74 ± 0.44	0.061

Values are presented as mean ± standard deviation, or number (%). *p*-values were calculated using the Chi-square test or the Mann–Whitney U test. ^a^ *p*-value calculated by Fisher’s exact test. * *p* < 0.05, ** *p* < 0.01, *** *p* < 0.001. Abbreviations: BMI, body mass index; CRP, C-reactive protein; dBP, diastolic blood pressure; GAD, generalized anxiety disorder; HR, heart rate; sBP, systolic blood pressure; PHQ, patient health questionnaire; VAS, visual analogue scale; WBC, white blood cell.

**Table 3:** Baseline demographic and clinical characteristics of patients with LSS assigned to GEO or PC inhalation as a function of pain severity.

Variable	Mild Pain			Moderate to Severe Pain		
	PC (n = 7)	GEO (n = 9)	*p*-Value	PC (n = 21)	GEO (n = 20)	*p*-Value
Pain-VAS, cm	2.47 ± 0.72	2.23 ± 0.69	0.470	5.59 ± 1.28	6.65 ± 2.05	0.105
Gender, women, n (%)	5 (71.43)	5 (55.56)	0.633 ^a^	14 (66.67)	18 (90.00)	0.130
Age, yr	67.57 ± 6.85	62.00 ± 9.66	0.185	66.33 ± 6.13	66.89 ± 6.01	0.354
BMI, kg/m^2^	25.82 ± 3.01	25.23 ± 2.25	0.560	61.16 ± 3.24	24.63 ± 3.33	0.705
Cigarette smoker, n (%)	1 (14.29)	1 (11.11)	1.000 ^a^	5 (23.81)	2 (10.00)	0.663 ^a^
Alcohol drinker, n (%)	1 (14.29)	3 (33.33)	0.585 ^a^	4 (19.05)	3 (15.00)	0.697 ^a^
Medications for pain control						
NSAIDs, n (%)	5 (71.43)	7 (77.78)	1.000	14 (66.67)	14 (70.00)	1.000
Opioids, n (%)	1 (14.29)	2 (22.22)	1.000 ^a^	7 (33.33)	7 (35.00)	1.000
Pregabalin, n (%)	3(42.86)	2 (22.22)	0.596 ^a^	6 (28.57)	5 (25.00)	1.000
Psychological status						
GAD-7, point	5.71 ± 6.50	5.11 ± 3.70	0.142	8.81 ± 4.41	8.15 ± 6.41	0.214
PHQ-9, point	7.43 ± 6.00	7.22 ± 4.06	0.351	9.10 ± 3.96	10.65 ± 8.11	0.794
Anxiety-VAS, cm	1.63 ± 1.79	1.19 ± 1.33	0.681	4.02 ± 2.00	3.95 ± 3.59	0.434
Stress-VAS, cm	1.64 ± 1.88	2.50 ± 2.50	0.758	4.01 ± 1.81	5.48 ± 3.95	0.303
Depression-VAS, cm	1.84 ± 1.60	1.93 ± 2.12	0.918	4.20 ± 2.01	3.71 ± 3.30	0.434
Hypertension, n (%)	5 (71.43)	4 (44.44)	0.358 ^a^	10 (47.62)	12 (60.00)	0.756
sBP, mmHg	135.14 ± 11.22	126.89 ± 11.50	0.152	128.76 ± 10.49	131.55 ± 13.58	0.896
dBP, mmHg	72.14 ± 6.44	74.78 ± 4.89	0.408	74.43 ± 10.76	74.90 ± 12.54	0.990
HR, bpm/min	80.86 ± 13.73	76.67 ± 15.26	0.606	78.10 ± 11.21	79.60 ± 11.35	0.583
Serum biochemical parameters						
WBC count, μL	5585.71 ± 1579.48	8422.22 ± 2008.59	0.120	6609.52 ± 1738.65	6690.00 ± 1784.41	0.774
CRP, mg/dL	1.40 ± 1.64	0.85 ± 0.82	0.606	1.19 ± 1.06	1.31 ± 1.50	0.845
Total Ca^2+^,mg/dL	10.14 ± 0.66	10.00 ± 0.59	0.536	9.82 ± 0.41	9.67 ± 0.47	0.260

Values are presented as mean ± standard deviation, or number (%). *p*-values were calculated using the Chi-square test or the Mann–Whitney U test. ^a^ *p*-value calculated using Fisher’s exact test. Abbreviations: BMI, body mass index; CRP, C-reactive protein; dBP, diastolic blood pressure; GAD, generalized anxiety disorder; GEO, geranium essential oil; HR, heart rate; sBP, systolic blood pressure PC, placebo control; PHQ, patient health questionnaire; VAS, visual analogue scale; WBC, white blood cell.

**Table 4:** Anxiety-VAS, stress-VAS, and depression-VAS scores of patients with LSS assigned to GEO or PC inhalation as a function of pain severity.

Variable	Mild Pain			Moderate To Severe Pain		
	PC (n = 7)	GEO (n = 9)	*p*-Value	PC (n = 21)	GEO (n = 20)	*p*-Value
Anxiety VAS, cm						
Pre	1.63 ± 1.79	1.19 ± 1.33		4.02 ± 2.00	3.95 ± 3.59	
Post	1.54 ± 1.44	0.76 ± 1.38		4.31 ± 1.82	2.87 ± 3.30	
Mean difference	−0.09 ± 0.43	−0.043 ± 1.16	0.681	0.29 ± 1.26	−1.08 ± 1.65	0.002 **
*p*-value	0.786	0.271		0.288	0.002 **	
Stress VAS, cm						
Pre	1.64 ± 1.88	2.50 ± 2.50		4.01 ± 1.81	5.48 ± 3.95	
Post	1.40 ± 1.53	0.88 ± 1.43		3.92 ± 1.70	3.41 ± 3.61	
Mean difference	−0.24 ± 0.43	−1.62 ± 2.51	0.408	−0.09 ± 1.35	−2.08 ± 2.21	0.003 **
*p*-value	0.039 *	0.043 *		0.823	>0.001 ***	
Depression VAS, cm						
Pre	1.84 ± 1.60	1.93 ± 2.12		4.20 ± 2.01	3.71 ± 3.30	
Post	1.81 ± 1.67	0.77 ± 1.19		4.16 ± 1.71	3.09 ± 3.33	
Mean difference	−0.03 ± 0.51	−1.17 ± 2.44	0.606	−0.05 ± 1.60	−0.62 ± 1.35	0.283
*p*-value	0.893	0.176		0.687	0.030 *	

Values are presented as mean ± standard deviation. *p*-values were calculated using the Wilcoxon signed rank test or the Kruskal–Wallis rank sum test. * *p* < 0.05, ** *p* < 0.01, *** *p* < 0.001. Abbreviations: dBP, diastolic blood pressure; GEO, geranium essential oil; HR, heart rate; sBP, systolic blood pressure; PC, placebo control group; VAS, visual analogue scale.

**Table 5:** Pain-related variables at baseline and after inhalation of geranium essential oil or placebo control.

Variable	PC (n = 28)	GEO (n = 29)	*p*-Value
Medications for pain control			
NSAIDs, n (%)	19 (67.86)	21 (71.41)	0.707
Opioids, n (%)	8 (28.57)	9 (31.03)	0.839
Pregabalin, n (%)	9 (32.14)	7 (24.14)	0.501
Pain VAS, cm			
Pre	4.81 ± 1.79	5.28 ± 2.70	0.632
Post	4.42 ± 1.82	3.87 ± 2.89	
Mean difference	−0.39 ± 0.80	−1.41 ± 1.90	0.003 **
*p*-value	0.300	<0.001 ***	
Anxiety VAS, cm			
Pre	3.42 ± 2.18	3.09 ± 3.31	0.167
Post	3.62 ± 2.10	2.21 ± 2.98	
Mean difference	0.20 ±1.11	−0.88 ± 1.52	0.004 *
*p*-value	0.401	0.001 **	
Stress VAS, cm			
Pre	3.42 ± 2.07	4.56 ± 3.79	0.518
Post	3.29 ± 1.98	2.62 ± 3.29	
Mean difference	−0.13 ± 1.19	−1.93 ± 2.27	0.001 **
*p*-value	0.443	<0.001 ***	
Depression VAS, cm			
Pre	3.61 ± 2.15	3.16 ± 3.06	0.240
Post	3.57 ± 1.96	2.37 ± 3.02	
Mean difference	−0.04 ± 1.40	−0.79 ± 1.74	0.165
*p*-value	0.700	0.006 **	
sBP			
Pre	130.36 ± 10.84	130.10 ± 12.95	0.836
Post	128.04 ± 11.26	124.86 ± 14.32	
Mean difference	−2.32 ± 5.14	−5.24 ± 7.50	0.099
*p*-value	0.012 *	0.002 **	
dBP			
Pre	73.86 ± 9.80	74.86 ± 10.66	0.755
Post	75.36 ± 10.65	72.55 ± 11.78	
Mean difference	1.50 ± 4.42	2.31 ± 8.12	0.019 *
*p*-value	0.084	0.155	
HR, bpm/min			
Pre	78.79 ± 12.59	78.69 ± 12.48	0.905
Post	78.57 ± 11.99	77.03 ± 11.27	
Mean difference	−0.21 ± 4.75	−1.66 ± 6.60	0.350
*p*-value	0.799	0.074	

Values are presented as mean ± standard deviation. *p*-values were calculated using the Wilcoxon signed rank test or the Kruskal–Wallis rank sum test. * *p* < 0.05, ** *p* < 0.01, *** *p* < 0.001. Abbreviations: dBP, diastolic blood pressure; GEO, geranium essential oil; HR, heart rate; sBP, systolic blood pressure; PC, placebo control; VAS, visual analogue scale.

## Data Availability

Data is contained within the article.

## Supplementary Materials

The following supporting information can be downloaded at: https://www.mdpi.com/article/10.3390/ph17010001/s1, Figure S1. Representative chromatogram of Pelargonium graveolens essential oil.

## References

[1] Ravindra V.M., Senglaub S.S., Rattani A., Dewan M.C., Härtl R., Bisson E., Park K.B., Shrime M.G. (2018). Degenerative Lumbar Spine Disease: Estimating Global Incidence and Worldwide Volume. Glob. Spine J..

[2] Lee C.H., Chung C.K., Kim C.H., Kwon J.W. (2018). Health Care Burden of Spinal Diseases in the Republic of Korea: Analysis of a Nationwide Database from 2012 through 2016. Neurospine.

[3] Özdemir E., Paker N., Bugdayci D., Tekdos D.D. (2015). Quality of life and related factors in degenerative lumbar spinal stenosis: A controlled study. J. Back Musculoskelet. Rehabil..

[4] Hong J.H., Lee M.Y., Jung S.W., Lee S.Y. (2015). Does spinal stenosis correlate with MRI findings and pain, psychologic factor and quality of life?. Korean J. Anesthesiol..

[5] Katz J.N., Zimmerman Z.E., Mass H., Makhni M.C. (2022). Diagnosis and Management of Lumbar Spinal Stenosis: A Review. JAMA.

[6] Raja S.N., Carr D.B., Cohen M., Finnerup N.B., Flor H., Gibson S., Keefe F.J., Mogil J.S., Ringkamp M., Sluka K.A. (2020). The revised International Association for the Study of Pain definition of pain: Concepts, challenges, and compromises. Pain.

[7] Heikkinen J., Honkanen R., Williams L., Leung J., Rauma P., Quirk S., Koivumaa-Honkanen H. (2019). Depressive disorders, anxiety disorders and subjective mental health in common musculoskeletal diseases: A review. Maturitas.

[8] Pomara N., Lee S.H., Bruno D., Silber T., Greenblatt D.J., Petkova E., Sidtis J.J. (2015). Adverse performance effects of acute lorazepam administration in elderly long-term users: Pharmacokinetic and clinical predictors. Prog. Neuro-Psychopharmacol. Biol. Psychiatry.

[9] Minetama M., Kawakami M., Teraguchi M., Kagotani R., Mera Y., Sumiya T., Nakagawa M., Yamamoto Y., Matsuo S., Koike Y. (2019). Supervised physical therapy vs. home exercise for patients with lumbar spinal stenosis: A randomized controlled trial. Spine J..

[10] Violante F.S., Mattioli S., Bonfiglioli R. (2015). Low-back pain. Handb. Clin. Neurol..

[11] Luo Y., Wang C.Z., Sawadogo R., Tan T., Yuan C.S. (2020). Effects of Herbal Medicines on Pain Management. Am. J. Chin. Med..

[12] Tabatabaeichehr M., Mortazavi H. (2020). The Effectiveness of Aromatherapy in the Management of Labor Pain and Anxiety: A Systematic Review. Ethiop. J. Health Sci..

[13] Lin C.Y., Liao H.E., Change-Lee S.N., Yen Y.Y. (2022). Initial and Continuous Effects of Essential Oil Therapy in Relieving Knee Pain among Older Adults with Osteoarthritis. Altern. Ther. Health Med..

[14] Borgonetti V., López V., Galeotti N. (2022). Ylang-ylang (*Cananga odorata* (Lam.) Hook. f. & Thomson) essential oil reduced neuropathic-pain and associated anxiety symptoms in mice. J. Ethnopharmacol..

[15] Seol G.H., Jung M.H. (2011). Effect of Bergamot Essential Oil-Inhalation on Chronic Pain after Surgery for Lumbar Spinal Stenosis. J. Korean Biol. Nurs. Sci..

[16] Fekri N., El Amir D., Owis A., AbouZid S. (2021). Studies on essential oil from rose-scented geranium, *Pelargonium graveolens* L’Hérit. (Geraniaceae). Nat. Prod. Res..

[17] Rao B.R. (2009). Chemical composition and uses of Indian rose-scented Geranium (Pelargonium species) essential oil-A review. J. Essent. Oil Bear. Plants.

[18] Gazerani A., Sarchahi Z., Hosseini S.S., Abavisani M. (2021). The effect of inhalation aromatherapy of geranium on pain and physiological indices after appendectomy: A double-blind randomized clinical trial. Int. J. Surg. Open.

[19] Shirzadegan R., Gholami M., Hasanvand S., Birjandi M., Beiranvand A. (2017). Effects of geranium aroma on anxiety among patients with acute myocardial infarction: A triple-blind randomized clinical trial. Complement. Ther. Clin. Pract..

[20] Karimi N., Hasanvand S., Beiranvand A., Gholami M., Birjandi M. (2023). The effect of Aromatherapy with *Pelargonium graveolens* (*P. graveolens*) on the fatigue and sleep quality of critical care nurses during the COVID-19 pandemic: A randomized controlled trial. Explore.

[21] Rungqu P., Oyedeji O., Gondwe M., Oyedeji A. (2023). Chemical Composition, Analgesic and Anti-Inflammatory Activity of Pelargonium peltatum Essential Oils from Eastern Cape, South Africa. Molecules.

[22] Lizarraga-Valderrama L.R. (2021). Effects of essential oils on central nervous system: Focus on mental health. Phytother. Res..

[23] Yamamoto Y., Kawakami M., Minetama M., Nakagawa M., Teraguchi M., Kagotani R., Mera Y., Sumiya T., Matsuo S., Kitano T. (2022). Psychological Predictors of Satisfaction after Lumbar Surgery for Lumbar Spinal Stenosis. Asian Spine J..

[24] Minetama M., Kawakami M., Teraguchi M., Kagotani R., Mera Y., Sumiya T., Nakagawa M., Yamamoto Y., Matsuo S., Sakon N. (2022). Associations between psychological factors and daily step count in patients with lumbar spinal stenosis. Physiother. Theory Pract..

[25] Houle M., Bonneau J.D., Marchand A.A., Descarreaux M. (2021). Physical and Psychological Factors Associated with Walking Capacity in Patients with Lumbar Spinal Stenosis with Neurogenic Claudication: A Systematic Scoping Review. Front. Neurol..

[26] Leeuw M., Goossens M.E., Linton S.J., Crombez G., Boersma K., Vlaeyen J.W. (2007). The fear-avoidance model of musculoskeletal pain: Current state of scientific evidence. J. Behav. Med..

[27] Halicka M., Duarte R., Catherall S., Maden M., Coetsee M., Wilby M., Brown C. (2022). Predictors of Pain and Disability Outcomes Following Spinal Surgery for Chronic Low Back and Radicular Pain: A Systematic Review. Clin. J. Pain.

[28] Pereira R.B., Rahali F.Z., Nehme R., Falleh H., Jemaa M.B., Sellami I.H., Ksouri R., Bouhallab S., Ceciliani F., Abdennebi-Najar L. (2023). Anti-inflammatory activity of essential oils from Tunisian aromatic and medicinal plants and their major constituents in THP-1 macrophages. Food Res. Int..

[29] Su Y.W., Chao S.H., Lee M.H., Ou T.Y., Tsai Y.C. (2010). Inhibitory effects of citronellol and geraniol on nitric oxide and prostaglandin E_2_production in macrophages. Planta Med..

[30] Galea C. (2023). Perspectives on the use of geranium essential oil: Pelargonium graveolens and pelargonium roseum, in dental medicine. Rom. J. Med. Dent. Educ..

[31] Enthoven W.T., Roelofs P.D., Deyo R.A., van Tulder M.W., Koes B.W. (2016). Non-steroidal anti-inflammatory drugs for chronic low back pain. Cochrane Database Syst. Rev..

[32] Bampidis V., Azimonti G., Bastos M.L., Christensen H., Durjava M., Kouba M., López-Alonso M., López Puente S., Marcon F., Mayo B. (2023). Safety and efficacy of a feed additive consisting of an essential oil from the herbaceous parts of *Pelargonium graveolens* L’Hér. (geranium rose oil) for all animal species (FEFANA asbl). EFSA J..

[33] Abouhosseini Tabari M., Hajizadeh Moghaddam A., Maggi F., Benelli G. (2018). Anxiolytic and antidepressant activities of *Pelargonium roseum* essential oil on Swiss albino mice: Possible involvement of serotonergic transmission. Phytother. Res..

[34] Tesfaye S., Sloan G., Petrie J., White D., Bradburn M., Julious S., Rajbhandari S., Sharma S., Rayman G., Gouni R. (2022). Comparison of amitriptyline supplemented with pregabalin, pregabalin supplemented with amitriptyline, and duloxetine supplemented with pregabalin for the treatment of diabetic peripheral neuropathic pain (OPTION-DM): A multicentre, double-blind, randomised crossover trial. Lancet.

[35] Konno S., Oda N., Ochiai T., Alev L. (2016). Randomized, Double-blind, Placebo-controlled Phase III Trial of Duloxetine Monotherapy in Japanese Patients with Chronic Low Back Pain. Spine.

[36] Skljarevski V., Ossanna M., Liu-Seifert H., Zhang Q., Chappell A., Iyengar S., Detke M., Backonja M. (2009). A double-blind, randomized trial of duloxetine versus placebo in the management of chronic low back pain. Eur. J. Neurol..

[37] Park S.Y., An H.S., Moon S.H., Lee H.M., Suh S.W., Chen D., Jeon J.H. (2015). Neuropathic Pain Components in Patients with Lumbar Spinal Stenosis. Yonsei Med. J..

[38] Haddadi K., Asadian L., Isazade A. (2016). Effects of Nasal Calcitonin vs. Oral Gabapentin on Pain and Symptoms of Lumbar Spinal Stenosis: A Clinical Trial Study. Clin. Med. Insights Arthritis Musculoskelet. Disord..

[39] Bussières A., Cancelliere C., Ammendolia C., Comer C.M., Zoubi F.A., Châtillon C.E., Chernish G., Cox J.M., Gliedt J.A., Haskett D. (2021). Non-Surgical Interventions for Lumbar Spinal Stenosis Leading to Neurogenic Claudication: A Clinical Practice Guideline. J. Pain.

[40] Lee J.Y., Choi H.Y., Park C.S., Jang C., Lee K.T., Lee J.Y., Youn I., Yune T.Y. (2019). Inhibition of COX-2 alleviates lumbar spinal stenosis-induced chronic mechanical allodynia in rats. Int. Immunopharmacol..

[41] Nikaido T., Takatsuna H., Tabata S., Shiosakai K., Nakatani T., Konno S.I. (2022). Efficacy and Safety of Add-on Mirogabalin to NSAIDs in Lumbar Spinal Stenosis with Peripheral Neuropathic Pain: A Randomized, Open-Label Study. Pain Ther..

[42] Sairyo K., Biyani A., Goel V.K., Leaman D.W., Booth R., Thomas J., Ebraheim N.A., Cowgill I.A., Mohan S.E. (2007). Lumbar ligamentum flavum hypertrophy is due to accumulation of inflammation-related scar tissue. Spine.

[43] Zheng Z.Y., Li P., Ao X., Qian L., Peng Y.X., Chu J., Jiang T., Lian Z.N., Zhang Z.M., Wang L. (2021). Characterization of a Novel Model of Lumbar Ligamentum Flavum Hypertrophy in Bipedal Standing Mice. Orthop. Surg..

[44] Seol G.H., Kang P., Lee H.S., Seol G.H. (2016). Antioxidant activity of linalool in patients with carpal tunnel syndrome. BMC Neurol..

[45] Wendin K., Pálsdóttir A.M., Spendrup S., Mårtensson L. (2023). Odor Perception and Descriptions of Rose-Scented Geranium *Pelargonium graveolens* ‘Dr. Westerlund’-Sensory and Chemical Analyses. Molecules.

[46] Boonstra A.M., Schiphorst Preuper H.R., Balk G.A., Stewart R.E. (2014). Cut-off points for mild, moderate, and severe pain on the visual analogue scale for pain in patients with chronic musculoskeletal pain. Pain.

