# Supplementary figures and images for: Inhalation of Pelargonium graveolens Essential Oil Alleviates Pain and Related Anxiety and Stress in Patients with Lumbar Spinal Stenosis and Moderate to Severe Pain
Source: Pharmaceuticals (Basel). 2023 Dec 19;17(1):1. doi: 10.3390/ph17010001 (PMC10818376; doi:10.3390/ph17010001)

**Figure S1.** Representative chromatogram of *Pelargonium graveolens* essential oil

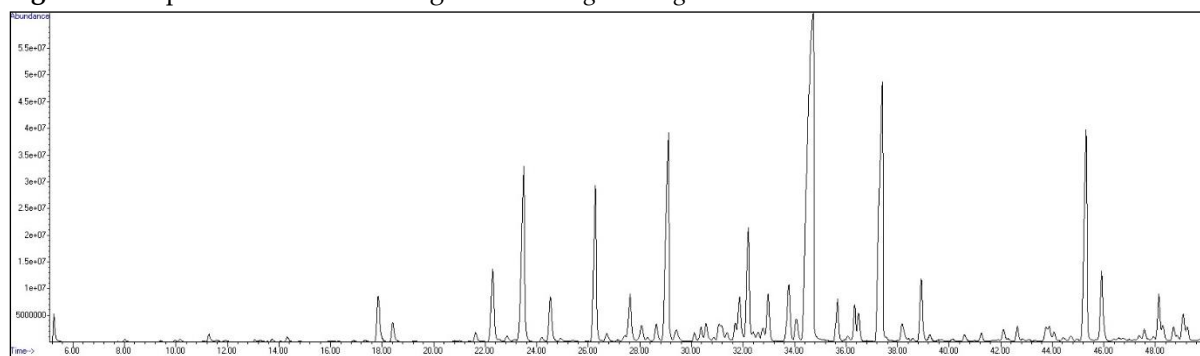

Supplement: Supplementary file 1 [file pharmaceuticals-17-00001-s001.zip › pharmaceuticals-2725301-supplementary.pdf]
